# CBD2023: A Hypercomplex Bangla Handwriting Character Recognition Data for Hierarchical Class Expansion

**DOI:** 10.1016/j.dib.2023.109909

**Published:** 2023-12-08

**Authors:** Jabed Omor Bappi, Mohammad Abu Tareq Rony

**Affiliations:** a Electrical and Electronic Engineering, Port City International University, Chittagong-4209, Bangladesh; b Department of Statistics, Noakhali Science and Technology University, Noakhali-3814, Bangladesh

**Keywords:** Handwriting Character, Recognition, Bangla, Enlarge, Deep Learning

## Abstract

Object recognition technology has made significant strides, but recognizing handwritten Bangla characters (including symbols, compound forms, etc.) remains a challenging problem due to the prevalence of cursive writing and many ambiguous characters. The complexity and variability of the Bangla script and individual's unique handwriting styles make it difficult to achieve satisfactory performance for practical applications, and the best existing recognizers are far less effective than those developed for English alpha-numeric characters. Compared to other major languages, there are limited options for recognizing handwritten Bangla characters. This research has described a new dataset to improve the accuracy and effectiveness of handwriting recognition systems for the Bengali language spoken by over 200 million people worldwide. This dataset aims to investigate and recognize Bangla handwritten characters, focusing on enlarging the recognized character classes. To achieve this, a new challenging dataset for handwriting recognition is introduced, collected from numerous students' handwriting from two institutions.

Specifications Table

**Table:** 

Subject	Computer Science, Machine learning
Specific subject area	Deep learning, NLP, Text-to-speech, Corpus
Data format	Raw data (.jpg or .png)
Type of data	Image type
Data collection	First, the Bangla words are written on an A4 size page, and some photocopy is provided to people. They report on another A4 size paper by following a photocopy. Most are students from Nazirhat Collegiate High School and Nazirhat College, especially those from Orbit Coaching Center.
Data source location	Nazirhat, Chittagong, Bangladesh
Data accessibility	Repository name: Mendeley Data Data identification number: 10.17632/p8988t5cwg.5 Direct URL to data: https://data.mendeley.com/datasets/p8988t5cwg/5

## 1 Value of the Data

• The Bangla Handwritten Character Recognition Dataset comprises 583 individual classes, with 49 representing primary Bangla characters. This dataset is valuable for tasks such as Optical Character Recognition (OCR) and various research endeavours in the Natural Language Processing (NLP) sectors mentioned in specification table. Machine learning researchers can employ these datasets to evaluate and benchmark the effectiveness of various algorithms when addressing similar problem types, such as image classification and Image Recognition.
• This dataset currently comprises handwritten samples from individuals labelled as college and high school students. However, its value and versatility can be enhanced by expanding its scope to include diverse professionals such as doctors, teachers, individuals from various professions, and a broader spectrum of students from primary and secondary to high school and university levels. This more comprehensive dataset would prove invaluable for addressing many significant challenges and applications.
• This dataset deliberately excludes certain characters in the Bengali script to maintain its manageability within Natural Language Processing (NLP). The selection process focuses on unique textures, particularly those incorporating the Bengali' kar-fola' (diacritical) marks on both sides, simplifying the dataset while preserving its linguistic richness. Though this research systematically describes new custom datasets, there is also a publicly available Bangla handwritten character dataset called the Grapheme dataset [1]. The improvements achieved in this data bridge a notable disparity between the practical needs and the actual performance of Bangla handwritten character recognition systems.

## 2 Background

Another dataset has provided a valuable resource for training and testing such models, offering a rich source of diverse handwritten characters [2]. Additionally, the BornoNet architecture-2, with its impressive results in Bangla character recognition, showcases the effectiveness of deep learning approaches in tackling complex scripts [3]. Furthermore, in another study, the innovative perspective that employs a fundamental point-based approach has shown promise for Bangla and various scripts [4]. Lastly, explicitly designed for Bangla and accommodating multilingual contexts significantly improves CNN-based handwritten character recognition [5]. Hypercomplex Bangla Handwriting, Character Recognition Dataset Contains all the possible unique formats of Bangla Characters. Numerous research studies have been conducted to recognize various forms of Bangla handwriting, encompassing primary characters, compound characters, special symbols, and numerical values [5]. However, most of these investigations have concentrated on a limited set of 50 primary characters or characters associated with numerical values. These papers collectively lay the groundwork for further research, driving the field towards more accurate and versatile character recognition systems [6]. This study emphasizes the Grapheme dataset and a newly curated custom dataset.

## 3 Data Description

Table 1 shows the number of the custom dataset that was checked and cleaned after collecting data, and a total of 75,542 train images and 8,554 test images were obtained, including vowels, numeric values, symbols with kar-fola, consonants, and compound characters.

**Table 1:** Train and Test data.

	Train	Test
**Sample**	75,542	8,554

This dataset comprises approximately 84,000 meticulously organized Bangla character images, serving as a valuable resource for research in Bangla character recognition. Featuring 583 distinct character classes, including numerical characters, it provides a comprehensive foundation for researchers exploring various machine learning algorithms, developing deep neural networks, or conducting comparative studies in the field.

To generate this dataset, Bangla words were initially written on A4-sized pages. Photocopies of the text were distributed to individuals, primarily students from Nazirhat Collegiate High School and Nazirhat College. Participants reproduced the text on another A4-sized paper based on the photocopy. The resulting dataset is organized as a zip file containing two main folders: “train data” and “test data.”

**Train data:** Together Folder: This folder contains all images without labels. The corresponding labels and image names are stored in a CSV file called “full_df_train.csv.” The CSV file has three columns: “image_name,” “Label,” and “long_label.” The “Label” column categorizes classes into broader groups such as ‘consonants,’ ‘vowels,’ ‘compounds,’ ‘numbers,’ ‘kar-fola,’ etc. The “long_label” column provides more detailed labels, with 583 individual classes denoted by numbers like 1, 2, 3, 4, etc.

Total_datafinal Folder: This folder is independent and contains 583 subfolders, each representing a distinct class. Images within each subfolder correspond to the respective class name. Unlike the “Together” folder, the images here are labeled independently, making them suitable for different use cases. The “long_label” in the “Together” folder and the subfolder names in this folder are identical.

**Test data:** Test_Datatogether Folder: Similar to the “Together” folder in the training data, this folder contains images without labels. The corresponding labels and image names are stored in a CSV file connected to it. Test_Data Folder: This folder is independent and mirrors the structure of the “Total_datafinal” folder in the training set. It consists of 583 subfolders, each representing a class, with images stored accordingly. The meticulous organization ensures the dataset's usability and accessibility for various research applications in the realm of Bangla character recognition. Researchers can leverage this dataset for tasks such as Bangla character recognition, with the flexibility to use the labeled or unlabeled versions based on their specific requirements.

**Figure 1:**
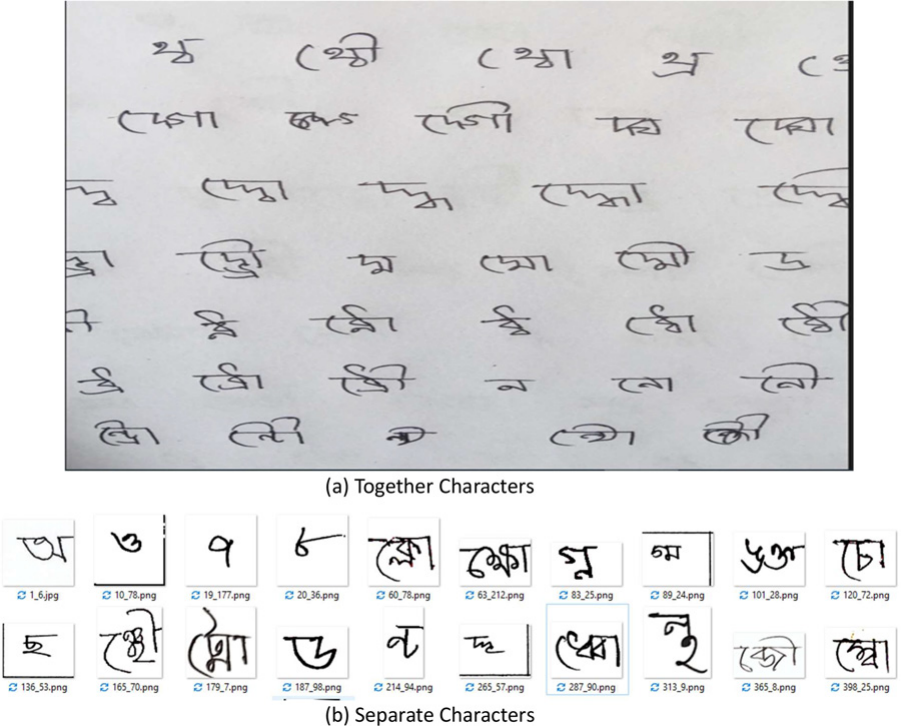
Prepare the dataset (a) Together Characters. (b) Separate Characters.

In Bengali, the terms a “kar” and “fola” refer to distinct features associated with consonant characters. The former denotes the vowel sound accompanying a consonant, represented by a diacritic mark added to the consonant character, while the latter refers to components also represented by diacritic marks added to consonant characters. The handwritten pages underwent meticulous digital capture and cropping, resulting in individual images for each class, as illustrated in Fig. 1.

While the internet hosts a variety of data types, it's crucial to note that the maximum class count for the entire Bangla dataset is 221, excluding the Grapheme Dataset. The Grapheme dataset, a valuable open-source resource for recognizing handwritten Bangla characters, stands as a significant reference in our study [1]. It encompasses over 411,000 isolated Bangla character images, featuring 1295 unique classes, including ‘kar’ and ‘fola’. This dataset includes all Bangla alphabets, covering vowels, consonants, and compounds. The dataset is organized into three output sections, each dependent on the others. However, it's noteworthy that numeric values in the Bangla script are not part of this dataset.

Our investigation unveiled that certain Grapheme roots exclusively possess vowel diacritics without accompanying consonant diacritics. The projected unique class count for the Grapheme dataset reaches 14,784 (168 × 11 × 8) by multiplying these three diacritic elements. Recognizing that specific class combinations may lack practical relevance within the Bengali language is essential. To summarize the key distinctions:

• Unlike the Grapheme dataset, which exclusively focuses on alphabetic characters, this custom dataset comprises both alphabetic and numeric characters.
• The Grapheme dataset's higher number of classes, compared to this custom dataset, may result in increased computational complexity and potentially lower accuracy.
• Precise accuracy calculation in the Grapheme dataset requires considering three diacritics, a contrast to the simpler structure of this custom dataset.
• While the Grapheme dataset represents each letter with a single character or alphabet, the custom dataset provides two different formats for certain alphabets.
• Notably, this custom dataset includes some alphabets that are absent in the Grapheme dataset.

**Figure 2:**
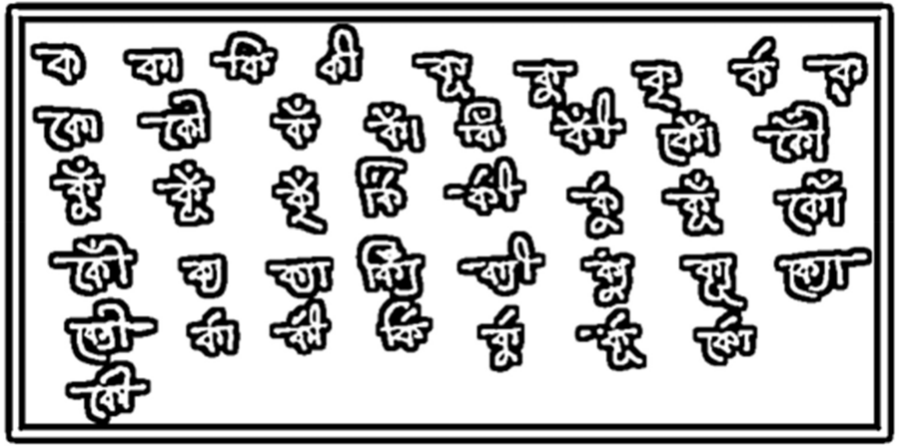
Multiple formats of one character.

The Bangla language encompasses over 11 variations of kar and fola significantly influencing the visual representation of its characters. Considering all possible combinations for each character would lead to an extensive number of classes for classification. For example, as depicted in Fig. 2, a single character may manifest up to 41 different formats due to the utilization of kar and fola. Moreover, the complexity can escalate when multiple kar and fola or both are applied to a single character. Surprisingly, no previous model has been designed to effectively recognize all the fundamental and compound characters encompassing these diverse variations. In response to this challenge, this dataset introduces a novel and demanding dataset for handwriting recognition. This dataset incorporates myriad variations arising from the nuanced application of kar and fola.

In developing the dataset, our primary goal was to reduce the potential number of classes. We noted that a single consonant has the capacity to generate up to 41 different characters. Excluding kar and fola, the Bangla language comprises 171 compounds, 33 consonant characters, 11 vowel characters, and 10 numeric values. It's essential to highlight that numerics and vowels do not intertwine with kar and fola. The calculation for probable classes involves adding the counts of compounds and consonants, multiplying the result by 41, and subsequently adding the number of vowel characters and numeric values. This computation results in a total of 8385 probable classes. While a character can exhibit up to 41 formats with the inclusion of all kar and fola, the majority of kars typically appear on either the left or right side of the character. Consequently, the overall probable class count is given by (((171+33) *41) +11+10) = 8385. Implementing such an approach would render the model more complex than other handwriting recognition models, posing challenges in dataset preparation. We grouped kar-fola and handwritten digits into a separate class to simplify the dataset. For instance, when the ‘ক’ state is in the middle and ‘ো’ kar or ‘ৌ’ kar is positioned on both sides of the alphabet, represented as ‘কো’ and ‘কৌ,’ we treated these instances as two distinct classes to ensure accurate image classification. Consequently, one alphabet can now have three formats. Following the reduction of classes, we arrived at 583 classes. This customised dataset has established two output sections, associating each character with two distinct output sections. A single character can thus have two separate output labels or classes. It's important to note that output section one encompasses 583 classes, whereas output section two comprises 5 classes. Output section two delineates the relationships and groupings among these 5 classes, while output section one provides individual classification labels for the 583 classes.

Table 2 outlines details about a comprehensive dataset comprising diverse Bangla characters, categorized into five distinct classes: Vowel, Consonant, Compound, Numeric, and Kar-Fola. The Vowel category comprises 11 samples, exemplifying characters such as অ, আ, and ই. Moving on to the Consonant category, it encompasses 107 samples. Notably, while standard Bangla has 39 consonants, our dataset considers each consonant in three different formats, leading to the count being three times greater (107 = 39 * 3). This category showcases a diverse array of characters and compound forms. The Compound category includes 441 samples, illustrating combinations of consonants. These combinations provide a nuanced representation of compound characters. In the Numeric category, 10 samples represent digits ranging from ০ to ৯. This category adds a numeric dimension to the dataset. Lastly, the Kar-Fola category features 11 samples, highlighting diacritic characters. These diacritics, known as Kar-Fola, play a crucial role in altering the pronunciation and appearance of characters. This diverse dataset is designed to capture the richness and complexity of Bangla characters in various forms and configurations, providing a valuable resource for character recognition and analysis.

**Table 2:** Custom dataset.

## 4 Experimental Design, Materials and Methods

**Figure 3:**
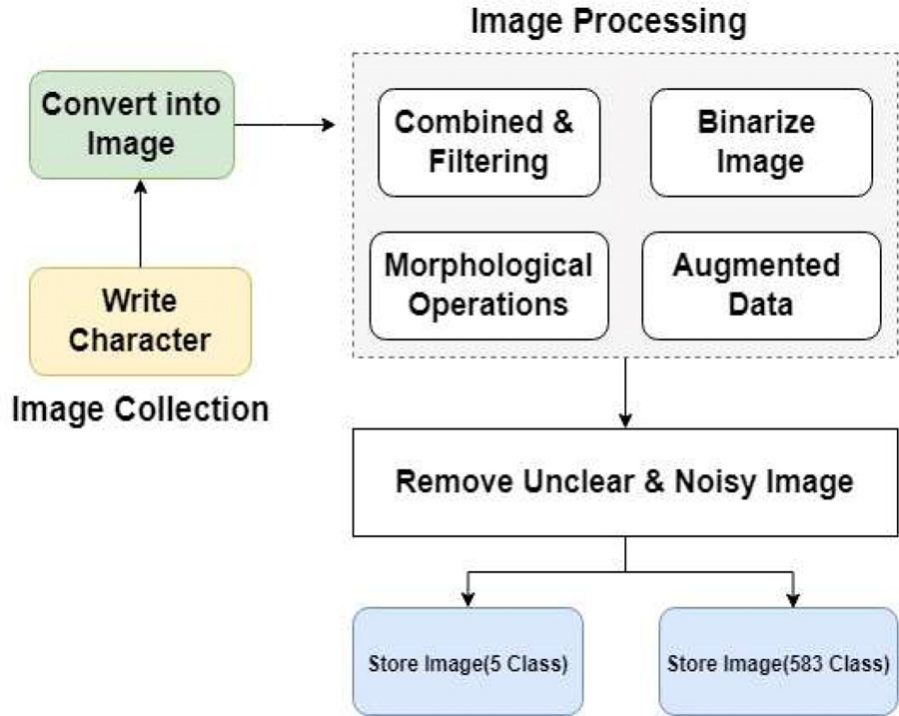
Data preparation and pre-processing.

Fig. 3 presents a comprehensive procedural overview for collecting and processing images, tailored to the intricacies of Bangla languages with their diverse and closely related alphabets. The diagram is structured into two principal sections: image collection and image processing, each serving distinct purposes in generating a robust dataset.

• **Character Writing:** This initial phase involves manually writing characters. This step ensures the inclusion of diverse writing styles and variations.
• **Converting Written Characters into Images (Representing a Single Class):** Following character writing, then converted into digital images. Each image corresponds to a single class, capturing the unique characteristics of the handwritten characters.

**Image Processing:** The image processing section comprises several sequential steps to enhance the quality and diversity of the dataset as follows;

• **Combination of Images into a Single Directory:** All collected images are stored in a unified directory. This step facilitates efficient handling and processing of the dataset as a whole.
• **Apply Filtering Techniques:** Filtering techniques are applied to refine the dataset. This may involve the removal of noise that could impact the accuracy.
• **Data Augmentation:** Data augmentation techniques enrich the dataset and enhance its variability. This step involves creating modified versions of existing images, such as rotation, scaling, or flipping, thereby expanding the datasets.
• **Convert Binary Format:** The processed images are converted into binary format. This step is crucial for standardizing the image representation and eliminating any ambiguity present in noisy or unclear images.
• **Storage in Separate Directories for Two Output Sections:** The final processed images are organized and stored in two distinct directories, aligning with the two output sections defined for the dataset. This structured storage ensures clarity and ease of access during subsequent stages of analysis.

**Figure 4:**
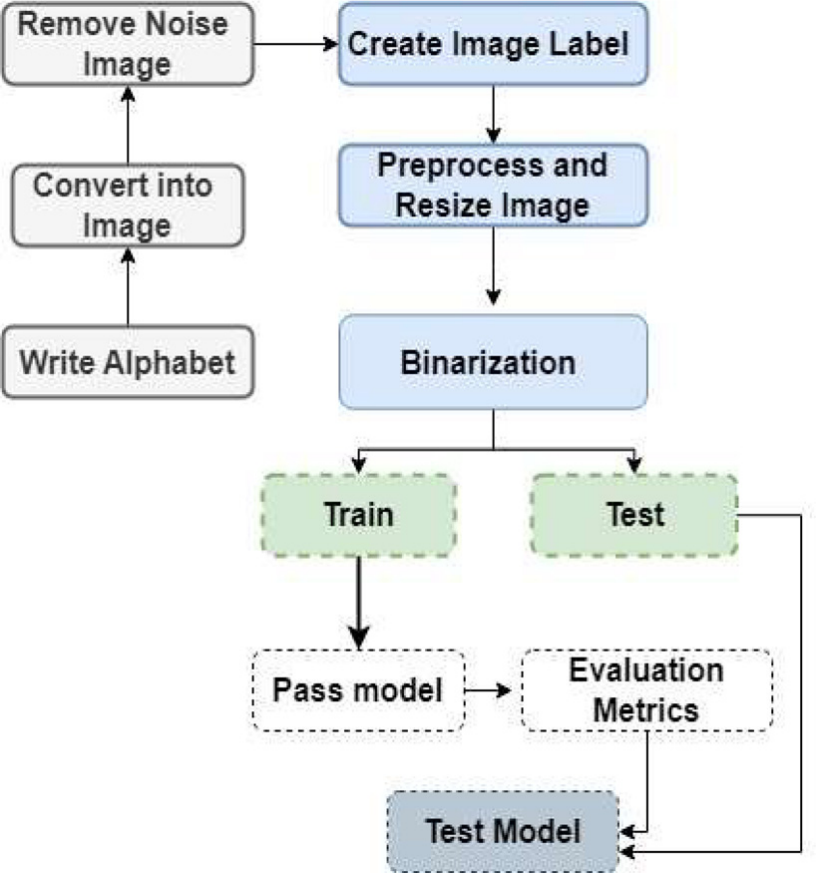
The development process of the CBD2023 dataset.

In Fig. 4 represents the development process of the dataset. The progression encompasses key phases aimed at creating a robust dataset for effective model training and evaluation.

• **Dataset Acquisition:** The initial phase involves the collection of a diverse set of alphabets. These characters are sourced from different individuals who have handwritten them on specific papers.
• **Labelling and Image Transformation:** Once the alphabets are obtained, corresponding labels are assigned to each image. Subsequently, these handwritten alphabets are transformed into digital images.
• **Noise Removal and Resizing:** To ensure the dataset's quality, noise removal techniques are applied, eliminating unwanted noise. Additionally, images are resized to a standardized dimension, ensuring uniformity across the dataset. This pre-processing step enhances image quality and prepares the dataset for subsequent analysis.
• **Binarization Procedure:** The grayscale images undergo binarization using a specified threshold value. This conversion transforms the images into a binary format, a crucial step for subsequent analysis and model training. Binarization simplifies image representation, making it easier for the model to identify patterns and features.
• **Dataset Division - Training and Testing Images:** After Completing the Image Binarization, the dataset is then divided into training and testing images.
• **Model Training:** The CNN model is trained using the training images, enabling it to grasp the underlying patterns and relationships within the dataset.
• **Model Testing:** Following training, the model is tested using the remaining images from the dataset. The model's effectiveness is evaluated based on its ability to classify and predict the test images' labels accurately.

## Limitations

*‘Not applicable’.*

## Ethics Statement

The authors have read and followed the ethical requirements for publication in Data in Brief and confirm that the current work does not involve human subjects, animal experiments, or any data collected from social media platforms.

## CRediT Author Statement

**Jabed Omor Bappi**: Methodology, Software, Validation, and Conceptualization –original draft preparation; **Mohammad Abu Tareq Rony**: Visualization, Writing, Investigation Supervision, Reviewing, and Editing.

## Data Availability

CBD2023:A Hypercomplex Bangla Handwriting Character Recognition Dataset for Hierarchical Class Expansion Using Deep Learning (Original data) (Mendeley Data)

## Appendix Supplementary materials

Image, application 1

Image, application 2
